# 3D-Printed Alginate Dialdehyde (ADA)–Gelatin (GEL) Hydrogels with Gallic Acid (GA) for Enhanced Multifunctional Properties

**DOI:** 10.1021/acsomega.6c02404

**Published:** 2026-06-02

**Authors:** Caroliny Oliveira Cavalcante, Hannah Sophia Kissel, Andreea Luiza Mîrt, José Yago R. Silva, Severino Alves Júnior, Aldo R. Boccaccini

**Affiliations:** † Departamento de Química Fundamental, Universidade Federal de Pernambuco, Cidade Universitária, 50740-560 Recife, PE, Brazil; ‡ Institute of Biomaterials, 9171 Friedrich-Alexander University Erlangen-Nuremberg, 91058 Erlangen, Germany

## Abstract

This study investigates the incorporation of gallic acid (GA) into alginate dialdehyde–gelatin (ADA–GEL) at GA concentrations (0.125%, 0.25%, and 0.5%). Spectroscopic analyses confirmed GA integration within the matrix, with Raman spectroscopy revealing GA vibrational bands (C–OH and C=C). Thermal and structural assessments indicated preservation of the polymer framework after GA incorporation, while scanning electron microscope (SEM) analysis showed increased surface roughness, suggesting network modification. GA functionalization enhanced phenolic content and antioxidant activity (∼96% DPPH radical scavenging) in films without significantly affecting swelling or degradation behavior, demonstrating maintained structural integrity. At 0.125% and 0.25% GA, improved rheological properties enabled successful three-dimensional (3D) printing of scaffolds with controlled pore size (∼0.6 mm). The printed scaffolds exhibited good cytocompatibility toward MC3T3-E1 preosteoblasts, while the films demonstrated antibacterial activity against *Staphylococcus aureus*, a pathogen associated with chronic bone infections. Overall, ADA–GEL–GA represents a promising biomaterial for bone tissue regeneration, combining structural stability, printability, and bioactive functionality.

## Graphical abstract

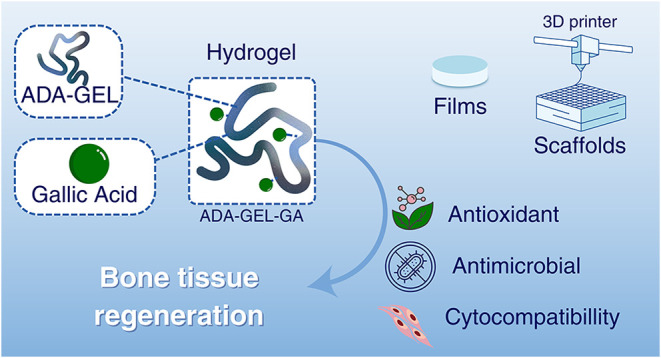

## Introduction

With the rapid progress of technology and the global increase in life expectancy, the continuous advancement of tissue engineering (TE) has become essential. Aimed at developing strategies for the prevention and treatment of traumatic injuries, TE seeks to minimize the negative effects on human health and overall quality of life.^1^ Current efforts focus on implementing supportive techniques with regenerative medicine, including the use of biocompatible materials designed to stimulate or regenerate damaged tissues.^2^ However, some patients may experience worsening of their clinical condition, increasing the risk of proliferation of pathogenic microorganisms such as bacteria, which take advantage of the fragile tissue and organs during the regeneration process.^3^ Currently, infections are commonly treated using topical or systemic antibiotics; however, such approaches present several limitations, including low drug concentration at the target site, the potential to induce microbial resistance, and undesirable side effects.^4^ These challenges highlight the need for the development of safer and more effective alternative therapeutic strategies, combining regenerative and anti-infection properties.

Phenolic compounds have emerged as valuable allies in combating bacterial infections and preventing cellular oxidative stress, acting as both antioxidant and antimicrobial agents.^5^ Moreover, they can enhance the efficacy of conventional drugs and support the recovery of damaged tissues. Gallic acid (GA) is a bioactive compound approved by the European Commission as a cosmetic ingredient with antioxidant function. In addition to being inexpensive, GA is a phenolic compound commonly found in *Camellia sinensis* (green tea), either in free form or associated with gallated tannins, and can be readily extracted at scale.^6^ It is also a key reagent for the synthesis of several industrial and pharmaceutical products, such as propyl gallate (approved by the U.S. FDA under 21 CFR 172.615) and tannic acid. GA has been extensively studied due to its antioxidant, anti-inflammatory, antiviral, antimicrobial, and antitumor properties.^7–9^ Structurally, GA contains an aromatic ring bearing three hydroxyl groups and one carboxylic acid, which enables multiple chemical interactions, high aqueous solubility, and strong affinity with polymeric matrices. Recently, GA has been incorporated into various biomaterial systems^10^ such as lysozymes,^11^ agarose,^12^ chitosan with hydroxyapatite,^13^ poly­(vinyl alcohol)^14^ to produce hydrogels giving them its intrinsic bioactivity and demonstrating promising results for wound treatment and bone regeneration.

Biopolymers derived from natural sources are widely accepted in regenerative medicine for the fabrication of injectable hydrogels.^15^ Among them, sodium alginate, derived from brown algae, stands out for its biocompatibility and its ability to form cross-linked networks in the presence of CaCl_2_, thereby reducing its high water absorption.^16^ The oxidation of alginate introduces aldehyde groups, leading to the formation of alginate dialdehyde (ADA), which preserves biocompatibility and enables stable chemical reactions, favoring hydrogel formation.^17^ Gelatin (GEL), a collagen-derived biopolymer, is another widely used natural material due to its bioabsorbable and biocompatible characteristics.^18^ The combination of ADA and GEL produces stable and printable hydrogels, formed through Schiff base (C=N) reactions between aldehyde and amide groups, providing a promising matrix for biomedical applications.^19^

Several research groups have explored strategies to enhance ADA–GEL hydrogels, by bioprintability with human umbilical vein endothelial cells^20^ and platelet-rich plasma^21^ or incorporating different functional components such as polyethylene glycol with borax,^22^ and Asiatic acid with borax for neural tissue.^23^ Our group has focused on both bone/musculoskeletal and wound/soft tissue regeneration. For bone applications, ADA–GEL has been modified with polydopamine,^24^ natural compounds such as ferulic acid,^25^ and inorganic salts like MgCO_3_.^26^ For soft tissue repair, formulations have included bioactive glasses,^27,28^ and nanosilicates.^29^ These additives have conferred new functionalities to the ADA–GEL system. Nevertheless, developing advanced materials for tissue regeneration remains challenging. The incorporation of gallic acid (GA) into the ADA–GEL matrix represents an alternative promising strategy to create printable hydrogels with antioxidant and antimicrobial properties, potentially useful for topical applications and enhanced drug delivery in cases of bacterial resistance or limited accessibility to the lesion site.

This study aims to develop for the first time ADA–GEL hydrogels containing different concentrations of gallic acid (0, 0.125, 0.25, and 0.5%), Figure 1. For this purpose, films were prepared and characterized regarding their phenolic content, antioxidant properties, GA release profile, degradation behavior, cytocompatibility with preosteoblast cells, and antimicrobial activity against *S. aureus* and *Escherichia coli*. Furthermore, the printability of these hydrogels and the fabrication of biocompatible scaffolds by 3D extrusion printing were evaluated. From the results obtained, we highlight for the first time the crucial role of gallic acid in its interactions with the ADA–GEL biopolymeric matrix, introducing antioxidant and antimicrobial properties and presenting a promising biomaterial technology for antibacterial tissue engineering.

**Figure 1:**
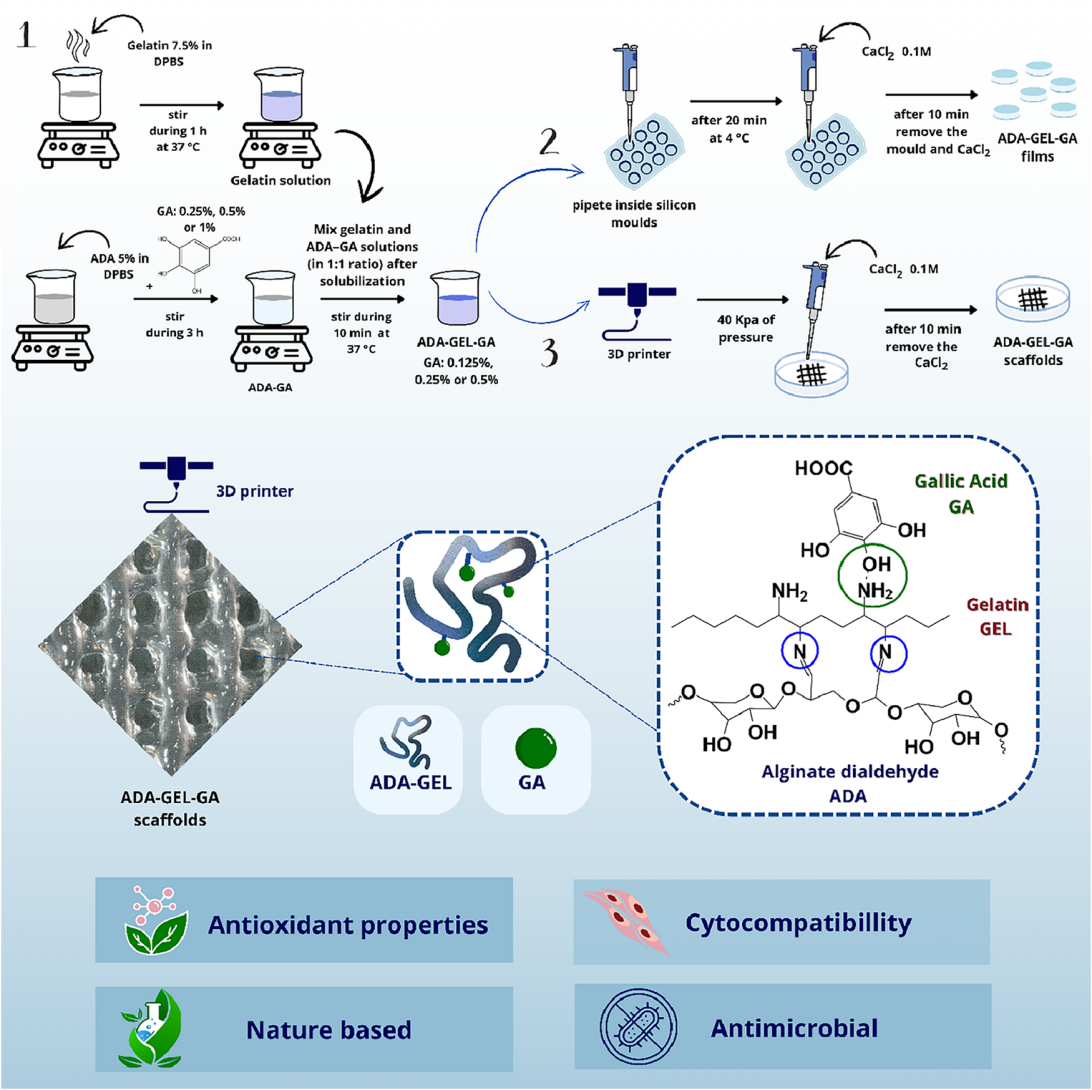
Schematic illustration of (1) synthesis steps, (2) film preparation, and (3) scaffold fabrication, including the structural outline of the ADA–GEL–GA system and its main functional properties.

## Results and Discussion

### Morphological and Physicochemical Properties

Hydrogels formed from alginate are widely studied in literature due alginate’s attractive characteristics such as being a biopolymer derived from natural sources, a nontoxic product, and easily accessible, enabling large-scale production.^30^ With the oxidation of alginate, dialdehyde alginate is obtained, in which aldehyde groups are formed, increasing the feasibility of interaction with other molecules, such as gelatin, through the formation of Schiff’s base (Figure 1, blue circle).^31^ The incorporation of phytotherapeutic compounds, particularly phenolic molecules, into hydrogels imparts valuable bioactive properties for tissue regeneration, including antioxidant and antibacterial activities.^10^ Aiming to obtain multifunctional hydrogels, dialdehyde alginate (ADA) and gelatin (GEL) were used as the polymer matrix, with gallic acid (GA) added in different concentrations. ADA-GEL-GA_*x*_ hydrogels were synthesized, where *x* = 0, 0.125%, 0.25%, or 0.5%. *x* = 0 corresponds to ADA-GEL. Hydrogels were prepared by mixing ADA 5% (w/v) with GA (0.25%, 0.5%, or 1%) (w/v) in Dulbecco’s Phosphate Buffered Saline (DPBS) and GEL 7.5% (w/v) in DPBS at a 1:1 ratio, Figure 1, forming transparent and homogeneous solutions.

To estimate the degree of oxidation, the proton Nuclear Magnetic Resonance (^1^H NMR) spectra of ADA were recorded in deuterated water (D_2_O) at 400 MHz. Since the region of interest overlaps with the residual solvent signal, an additional experiment with solvent suppression was performed to improve spectral resolution (Figure S1 a, b). According to Sanz-Horta et al. (2022), the relevant spectral window for determining the degree of oxidation lies between δ 4.90 and 6.00 ppm.^32^ In this range, the hemiacetal protons of ADA are observed at δ 5.40 and 5.70 ppm, while the signal at δ 4.95 ppm corresponds to H1-G, assigned to the anomeric proton (C1–H) of the guluronic acid (G) units in alginate.

In addition to the characteristic signals of ADA and alginate, integration of the peaks at δ 4.96 ppm (H1-G) and δ 5.43 ppm (hemiacetal protons) resulted in an approximate ratio of 1:0.13. This value is consistent with the degree of oxidation previously estimated using periodate-based oxidation, as reported by Karakaya et al., indicating approximately 13% oxidation.^19^

The interactions between the functional groups of ADA-GEL-GA_*x*_ were investigated using Fourier Transform Infrared Spectroscopy (FTIR), Figure 2a. GA exhibits characteristic bands of carboxylic acids, such as C=O and C–O stretching vibrations at 1664 and 1022 cm^–1^, respectively.^33^ ADA shows characteristic bands related to its polysaccharide structure, but the symmetric vibration of aldehyde at 1735 cm^–1^ is not detected, likely due to the equilibrium between aldehyde and hemiacetal groups within the ADA matrix, as previously reported in the literature.^34^ Additional evidence of alginate oxidation was obtained by ^1^H NMR through the detection of hemiacetal proton signals (Figure S1b). Notably, the asymmetric (COO^–^)_as_ and symmetric (COO^–^)_s_ carboxylate groups from ADA, at 1595 and 1408 cm^–1^, as well as the C–O–C stretching vibration at 1026 cm^–1^. In the GEL spectrum, the amide group (−CONH−) was identified by the amide I band, assigned to C=O stretching, at 1647 cm^–1^ and amide II band at 1541 cm^–1^, attributed to N–H in-plane bending coupled with C–N stretching.^35^ In the ADA-GEL spectrum, the amide I band shifted to 1622 cm^–1^, while the amide II band remained at 1541 cm^–1^, indicating interactions between gelatin amino groups and ADA aldehyde groups. These spectral changes may be associated with partial Schiff base formation, although the overlap between amide and imine vibrations prevents an unambiguous assignment of ν­(C=N).^26,36^ The ADA-GEL-GA_*x*_ (*x* = 0.125%, 0.25%, or 0.5%) hydrogels exhibited similar behavior to ADA-GEL, further supporting Schiff base formation. However, with the increase in gallic acid concentration, a decrease in the intensity of the band corresponding to the amide group (−CONH−) is observed, indicating a possible interaction through hydrogen bonding between the amide groups and the functional groups of gallic acid reducing their characteristic vibrational activity. On the other hand, characteristic bands of GA were not identified. The similarity between the functional groups of ADA and GA, particularly the carboxylic acid groups, may contribute to the masking of GA signals in the ADA-GEL-GA_*x*_ spectra due to band overlapping or the low GA concentration in the hydrogels.

**Figure 2:**
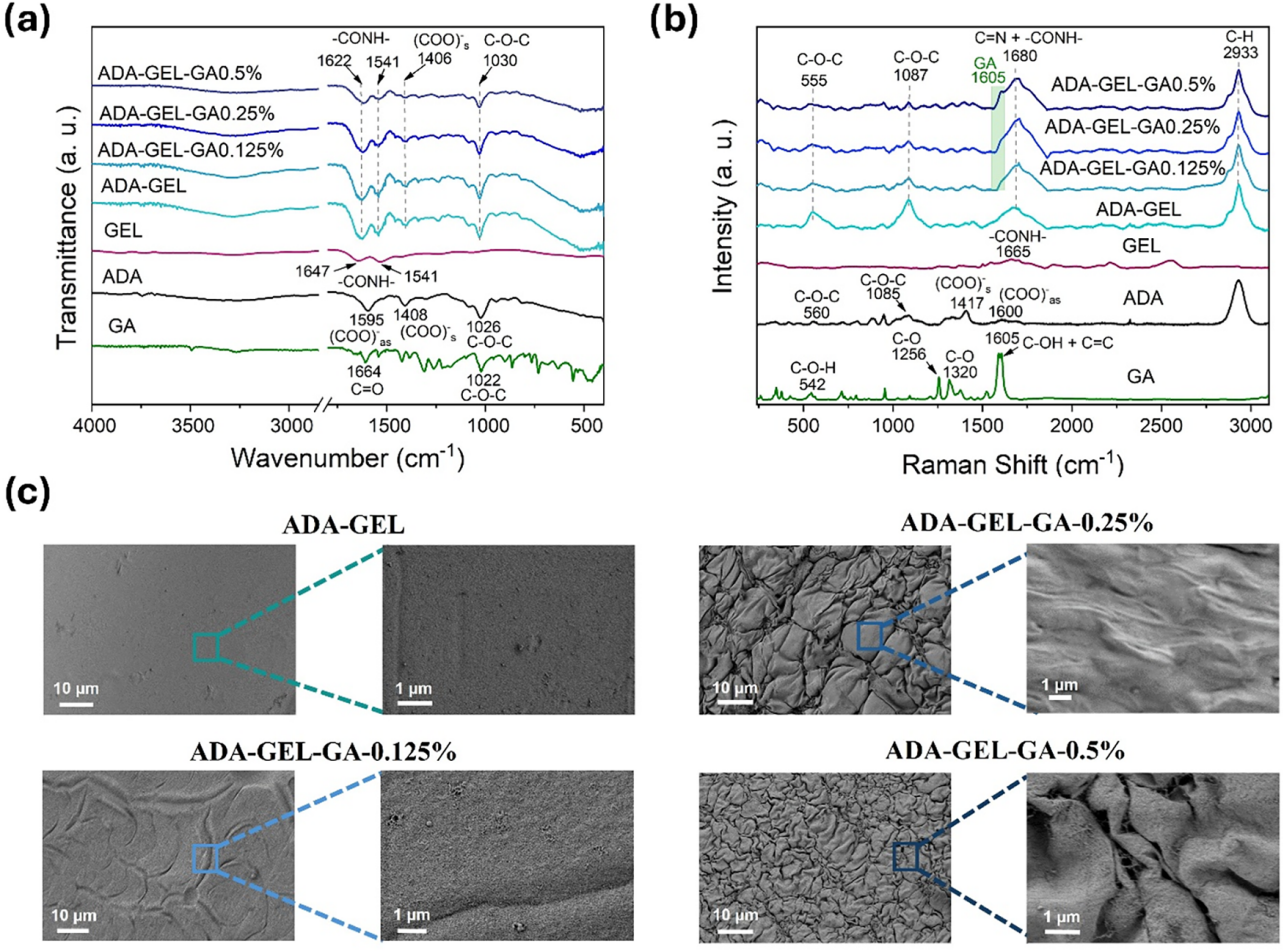
Spectroscopic and microscopic techniques (a) Fourier-transform infrared spectroscopy (FTIR) and (b) Raman spectroscopy of Gallic Acid (GA), alginate dialdehyde synthesis (ADA), gelatin (GEL), hydrogels (ADA-GEL, ADA-GEL-GA0.125%, ADA-GEL-GA0.25%, ADA-GEL-GA0.5%), (c) scanning electron microscopy (SEM) of hydrogels.

To complement the FTIR analysis, Raman spectroscopy was performed, Figure 2b. In the GA spectrum, a strong vibrational band at 1605 cm^–1^ was identified, attributed to aromatic C=C stretching and conjugated ν­(C=O) from the gallic acid. Additional bands include 1320 cm^–1^, assigned to conjugated C–O stretching coupled with aromatic ring (C=C) vibrations of gallic acid, 1256 cm^–1^, corresponding to out-of-plane C–O bending, and 542 cm^–1^ related to C–O–H deformation.^37^ The ADA spectrum displayed characteristic alginate bands, including C–H stretching at 2933 cm^–1^, asymmetric and symmetric carboxylate stretching (COO^–^)_a__s_ and (COO^–^)_s_ at 1600 and 1417 cm^–1^, respectively, as well as C–O–C stretching at 1085 cm^–1^ and C–O–C deformation at 555 cm^–1^, the latter associated with the oxidation of the M monomer in the polysaccharide backbone.^38^ For GEL, only the amide I band, attributed to C=O stretching of the –CONH– group, was observed at 1665 cm^–1^.^35^ In the ADA–GEL–GA_*x*_ spectra, characteristic ADA bands (C–O–C oxidized and C–O–C deformation) were retained, while a broad band centered at 1680 cm^–1^ was observed, which may be associated with interactions between gelatin and ADA, including possible Schiff base formation. This band likely results from the overlap between the – CONH– vibration of gelatin and the imine ν­(C=N) vibration.^39^ Additionally, an emerging shoulder at 1605 cm^–1^, whose intensity increased with higher GA concentration, corresponded to the C=C and conjugated ν­(C=O) vibrations of the phenolic group, confirming the presence of GA inside the ADA–GEL hydrogel matrix.

X-ray diffraction (XRD) analysis was performed, and the diffractograms revealed that pure GA exhibits a crystalline structure, which is not observed in the ADA-GEL-GA_*x*_ (*x* = 0.125%, 0.25%, or 0.5%) hydrogels, indicating their amorphous nature. Two broad peaks at 2θ = 31.8° and 45.6° were observed, which are attributed to intramolecular hydrogen bonds of sodium alginate (Figure S2).^25,31^

Thermogravimetric analysis (TGA) revealed similar thermal degradation profiles for all hydrogels (ADA-GEL, ADA-GEL-GA0.125%, ADA-GEL-GA0.25%, and ADA-GEL-GA0.5%) (Figure S3). A major weight loss of approximately 94.5% occurred below 170 °C, attributed to the dehydration of the hydrogel network.^31^ Differential thermal analysis (DTA) (Figure S4) showed a corresponding endothermic peak near 170 °C, confirming the solvent loss process. A subsequent exothermic event at approximately 500 °C was associated with the decomposition of organic matter, yielding residual masses of 5.0%, 5.12%, 5.9%, and 5.22% for ADA-GEL, ADA-GEL-GA0.125%, ADA-GEL-GA0.25%, and ADA-GEL-GA0.5%, (Figure S4b–e), respectively. No exothermic signal was detected around 270 °C, the typical degradation temperature of gallic acid (Figure S4a), likely due to its low content in the hydrogels, below the instrument’s detection limit. Nevertheless, the slight increase in organic residue relative to ADA-GEL supports the successful incorporation of gallic acid into the polymer matrix. All hydrogels also exhibited notable thermal stability.

To investigate the morphology of the materials, the films were dried using critical point drying (CPD) to preserve their surface integrity. Subsequently, scanning electron microscopy (SEM) was used to inspect the samples (Figure 2c). A progressive increase in surface roughness was observed with increasing gallic acid concentration, a phenomenon previously reported.^40^ This effect may be related to stronger interactions between the phenolic groups of gallic acid and the polymer matrix, leading to aggregation and surface heterogeneity.

Incorporation of gallic acid into the ADA–GEL matrix aimed to confer bioactivity arising from its phenolic nature, particularly antioxidant and antibacterial capabilities.^41^ The total phenolic content was subsequently evaluated using the Folin–Ciocalteu assay over a period of 7 days. A calibration curve of gallic acid (100–700 mg/L) was constructed (Figure S5a,b) to determine the phenolic concentration in the samples. As shown in Figure 3a, the total phenolic content increased proportionally with the gallic acid concentration incorporated into the hydrogels.

**Figure 3:**
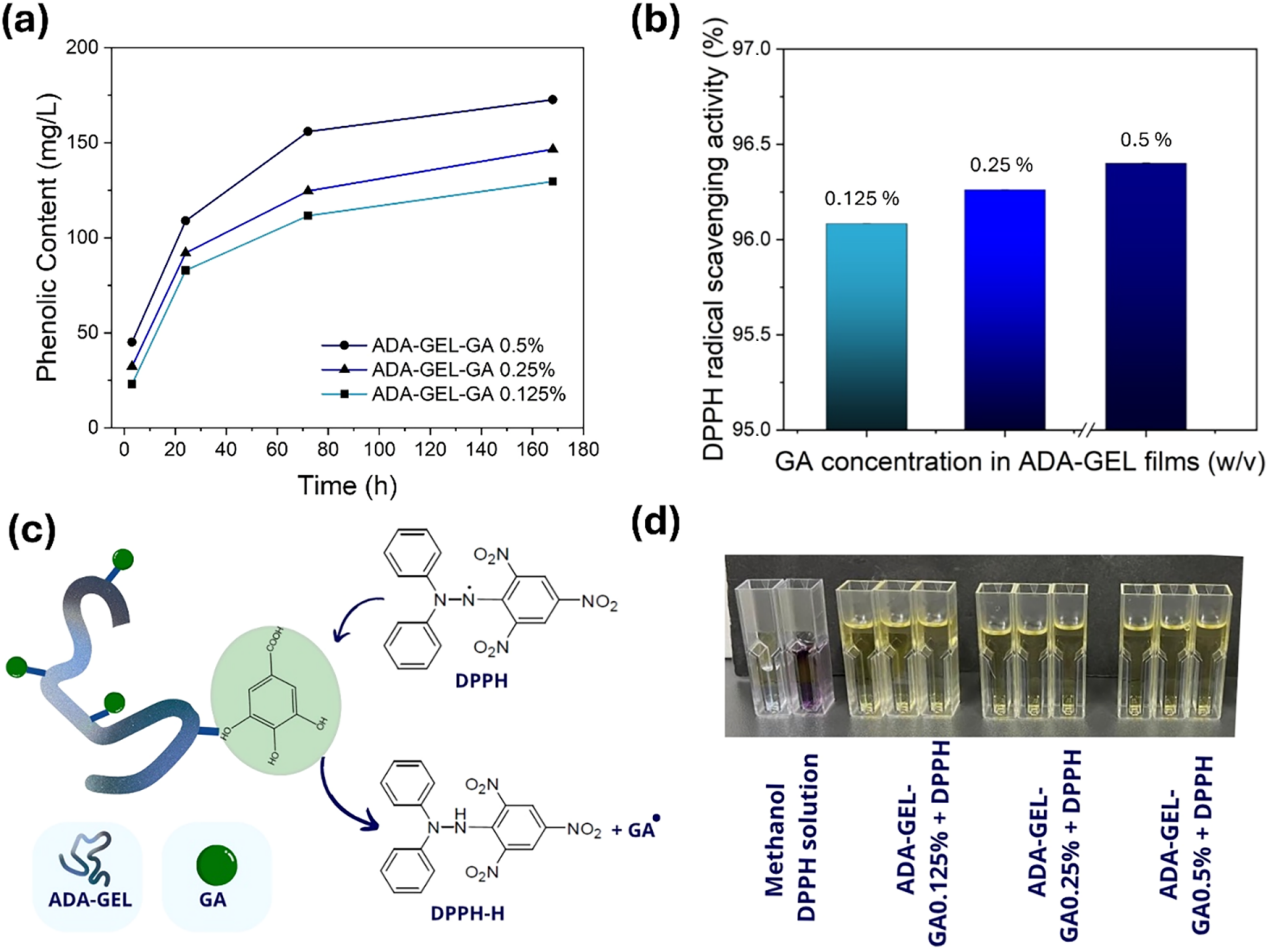
Antioxidant analysis of ADA–GEL–GA_*x*_ films (a) Phenolic content release; (b) DPPH radical scavenging activity; (c) Proposed mechanism of antioxidant action; and (d) cuvette images showing the antioxidant activity of ADA-GEL-GA_*x*_ films (*x* = 0.125%, 0.25%, and 0.5%) using the DPPH reagent.

The results from the high phenolic content had a direct impact on the antioxidant capacity of the polymer matrix.^42^ In the DPPH assay, the radical scavenging activity showed increasing values, reaching above 96% for ADA-GEL-GA_*x*_ films, where *x* = 0.125%, 0.25%, or 0.5%, Figure 3b. In this experiment, DPPH a stable free radical α reacts with phenolic groups, undergoing reduction to form DPPH-H, while the phenolic compound is oxidized to form a gallic acid radical, Figure 3c. This reaction also led to a visible color change, as shown in Figure 3d, where the characteristic purple color of DPPH shifted to yellow upon contact with the films.^43^ These excellent results outperform those reported for other polymer matrices incorporating gallic acid, such as chitosan-grafted 2-acrylamido-2-methylpropanesulfonic acid hydrogels,^33^ chitosan,^44^ GA grafted onto chitosan,^45^ and chitosan-interpenetrated p­(HEMA) hydrogels,^46^ while showing comparable performance to poly­(vinyl alcohol)-based systems.^47^

Swelling and degradation analyses were performed over short-term (up to 2 h, Figure 4a) and long-term (up to 27 days, Figure 4b) periods in cell medium to monitor the stability and degradation kinetics. All films exhibited similar swelling behavior, particularly within the first 2 h, during which the hydrogel expanded due to solvent diffusion, reaching approximately 60% for ADA-GEL-GA0.5%. This behavior is expected, since a higher number of −COOH groups enhances the formation of hydrogen bonds with water molecules, thereby increasing the hydrogel’s water uptake and swelling capacity.^33^ Degradation began after the first day for ADA-GEL,^19^ and by day 7, both ADA-GEL-GA0.5% and ADA-GEL films exhibited more pronounced degradation compared to ADA-GEL-GA0.125% and ADA-GEL-GA0.25% films. The similar behavior for all materials indicates that GA incorporation did not compromise the structural stability of the matrix.

**Figure 4:**
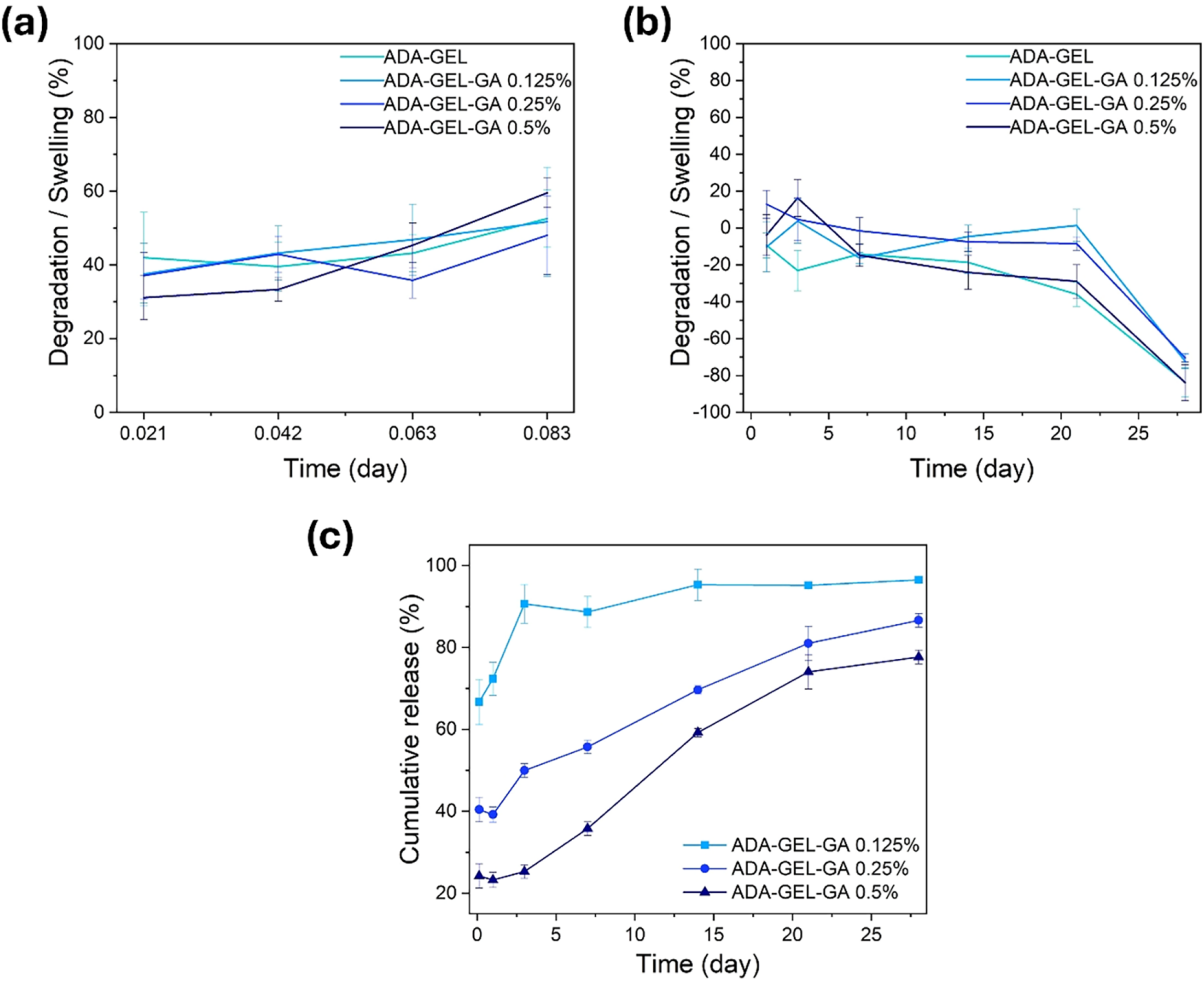
Swelling/degradation behavior of ADA-GEL-GA_*x*_ films (*x* = 0, 0.125%, 0.25%, and 0.5%) during (a) 30 min–2 h (b) 1–28 days and (c) Cumulative gallic acid (GA) release from ADA-GEL-GA_*x*_ films (*x* = 0.125%, 0.25%, and 0.5%) over 0.125–28 days.

To investigate the release kinetic of gallic acid, a release study was conducted in HBSS, a buffered solution with a pH of 7.4 commonly used for cell culture, as shown in Figure 4c. First, a calibration curve at 260 nm of Gallic acid was obtained, Figure S6, with *R* ^2^ = 0,997. At the initial time point (0.125 h), the concentration of released GA showed a higher proportion for the lower initial concentrations. On day 1, GA release reached approximately 23, 39, and 72%, for 0.5%, 0.25%, and 0.125% formulations, respectively, Figure 4c. The maximum release for 0.125% occurred on day 3, corresponding to about 90% of total GA, after that day the concentration remained plateaued between 95 and 97%. On day 7, GA0.25% films released about 56%, while for GA0.5% was 36%. After the 7 day, the 0.25% and 0.5% films continued to show a gradual increase in cumulative release until the end of the experimental period, indicating greater retention of the compound in the polymer matrix.

These differences in release percentages, where higher GA concentrations correspond to lower release ratios, may be attributed to several factors. At higher loadings, stronger intermolecular interactions, particularly hydrogen bonding between gallic acid and the ADA–GEL matrix, can restrict the diffusion of GA through the hydrogel network. In contrast, at lower concentrations, a more homogeneous distribution of GA within the matrix may facilitate faster and more complete release, also favored by increased swellability.^33^ However, based only on the swelling behavior, it is not possible to correlate the drug release kinetics of the films with the degree of swelling.

In order to investigate the impact of GA on ADA-GEL hydrogels, mechanical testing was performed using a microtester. Figure 5 shows the effective moduli of ADA-GEL-GA_*x*_ films measured directly after preparation. A progressive decrease in effective modulus was observed with increasing GA concentration. The pure ADA-GEL films were used as a reference and exhibited an effective modulus of 19.4 ± 3.8 kPa, which decreased to 17.7 ± 3.6 kPa for ADA-GEL-GA0.125%, 13.0 ± 4.9 kPa for ADA-GEL-GA0.25%, and 10.3 ± 3.7 kPa for ADA-GEL-GA0.5%. This reduction was statistically significant at the highest GA concentration (*p* < 0.05).

**Figure 5:**
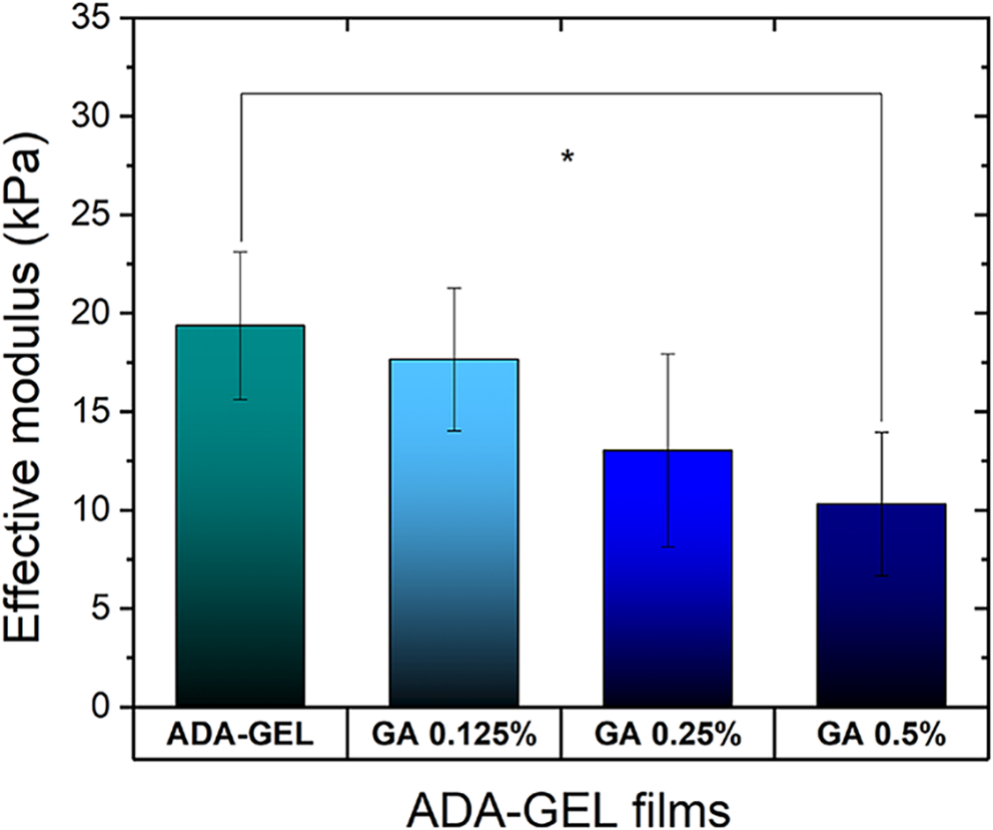
Effective modulus of ADA-GEL-GA_*x*_ films (*x* = 0.125%, 0.25%, and 0.5%).

This trend suggests that GA incorporation weakens the Ca^2+^-mediated ionic cross-linking network within the ADA phase. A plausible explanation is the formation of an acidic local microenvironment upon GA dissolution, which introduces additional H_3_O^+^ ions. Under such conditions, carboxylate groups (−COO^–^) on the ADA backbone become progressively protonated toward −COOH (p*K*_a_ of alginic acid reported between 3.6 for mannuronic acid and 3.8 for guluronic acid residues), reducing their availability for Ca^2+^ coordination.^48^ As a consequence, the stability of the characteristic “egg-box” junction zones is diminished due to partial disruption of Ca^2+^ bridges and ion exchange with protons. Notably, a potential reinforcing effect of GA on the GEL phase via protein–polyphenol interactions is unlikely to contribute under the present conditions, as such cross-linking pathways are primarily favored in alkaline conditions and are therefore not expected.^49^

From a bone tissue engineering perspective, the observed modulus range (approximately 10–20 kPa) remains several orders of magnitude lower than that of native cortical bone (in the GPa range), although it falls within the lower stiffness range reported for soft connective tissues.^50^ Therefore, ADA-GEL-GA hydrogels may be better suited as bioactive and cell-instructive matrices for biofabrication strategies, where they can provide a permissive microenvironment for cell encapsulation, proliferation, and early stage tissue formation rather than serving as load-bearing constructs.^51,52^

To initiate the 3D printing of scaffolds, it is essential to assess whether the materials exhibit a gelation point. All hydrogel formulations (ADA–GEL–GA_*x*_, where *x* = 0, 0.125%, 0.25%, and 0.5%) were initially tested using stainless steel needles with a tip diameter of 250 μm. The ADA-GEL-GA0.5% formulation did not demonstrate the filament formation required for scaffold fabrication, unlike the other formulations which successfully formed stable filaments, Figure 6a, ADA-GEL-GA_*x*_, where *x* = 0, 0.125% and 0.25%.

**Figure 6:**
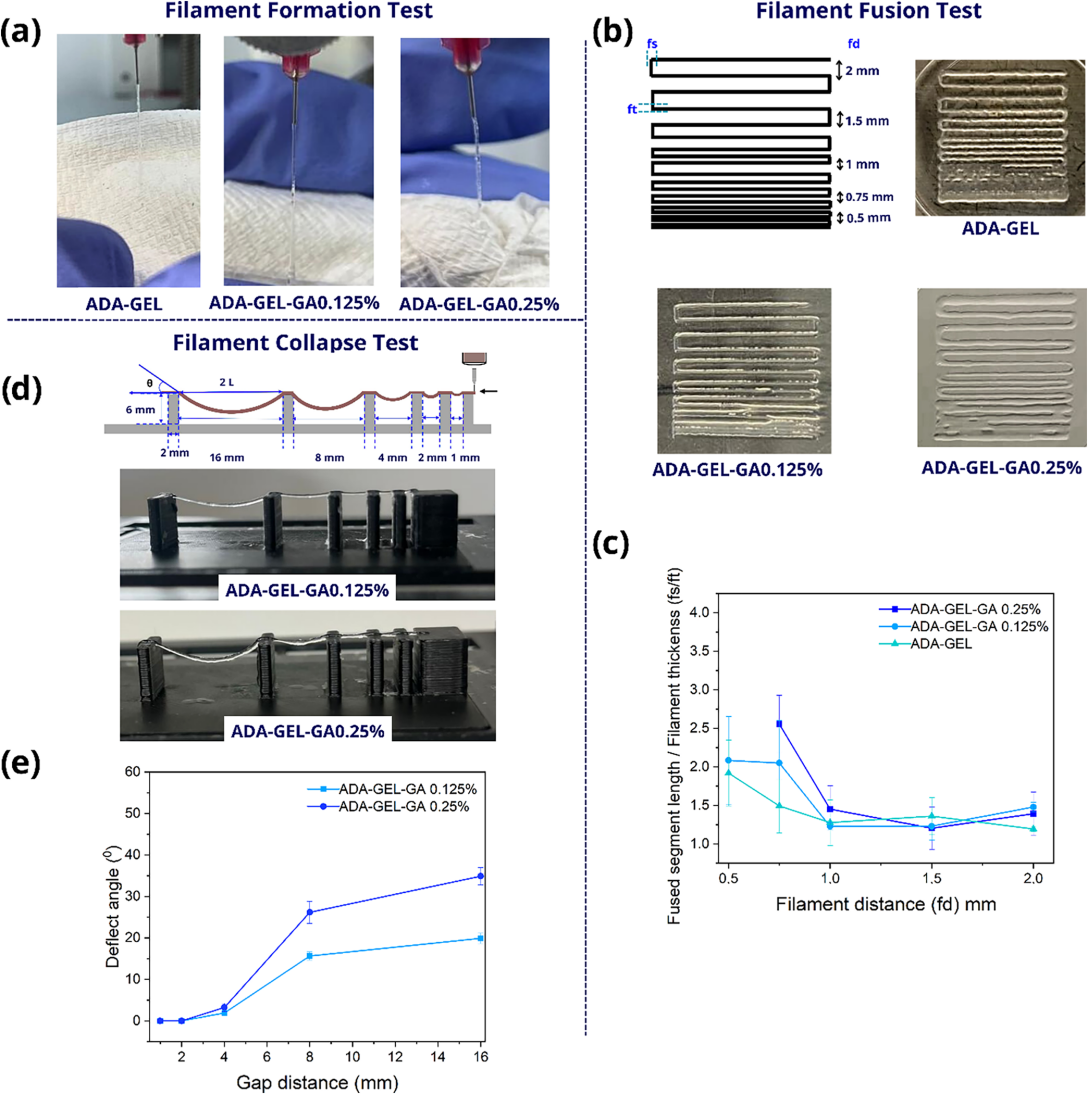
Printability tests of ADA-GEL-GA*_x_* films: (a) filament formation test; (b) filament fusion test; (c) plot of fused segment length and filament thickness for ADA-GEL-GA_*x*_ films (*x* = 0, 0.125%, and 0.25%); (d) filament collapse test; and (e) plot of deflection angle for ADA-GEL-GA_*x*_ films (*x* = 0.125% and 0.25%).

An important test to evaluate printability is the Filament Fusion Test (FFT), as it provides insights into the achievable printing resolution. The FFT was performed for ADA-GEL-GA_*x*_, where *x* = 0, 0.125%, and 0.25%, Figure 6b. Calculations were based on fixed filament distances (fd), using the measured fused segment length (fs) divided by the filament thickness (ft). As shown in Figure 6c, at distances between 1 and 2 mm, all hydrogels exhibited similar resolution values, with resolution ratios below 1.5. It was observed that increasing GA concentration resulted in a denser gel, which directly affected printing resolution. However, in the merging of the 0.5 mm and 0.75 mm lines for ADA-GEL-GA0.25%, it was not possible to measure the strand distances due to the filament fusion.

The Filament Collapse Test (FCT) was performed and revealed that the ADA-GEL-GA0.25% formulation exhibited a greater deflection angle compared to ADA-GEL-GA0.125%, Figure 6d, particularly at spanning distances between 4 and 16 mm, Figure 6e, indicating reduced structural stability and lower shape fidelity under unsupported printing conditions.

These results are consistent with mechanical tests, Figure 5, that showed less stiffness with increasing GA concentration. The formulation may be related to more extensive intermolecular interactions, including hydrogen bonding, π–π stacking, and hydrophobic interactions promoted by the presence of gallic acid.^45^ Similar effects have been reported in literature, where the incorporation of phenolic compounds into polymeric matrices promotes additional noncovalent interactions between hydroxyl or aromatic groups of phenolics and the polymer matrix, influencing rheological behavior and filament formation during printing.^25,53^ In this way, FFT and FCT demonstrated that ADA-GEL-GA0.125% and ADA-GEL-GA0.25% hydrogels (inks) could print well-defined and continuous filaments.

Subsequently, 3D printing of scaffolds was performed using ADA-GEL-GA_*x*_ hydrogels, where *x* = 0, 0.125% and 0.25%, as shown in the optical microscopy images, Figure 7a. Visually, subtle differences in pore morphology were observed among formulations: ADA-GEL exhibited more irregular pores; ADA-GEL-GA0.125% presented slightly more well-defined, square-like pores; and ADA-GEL-GA0.25% showed more rounded pores with lower structural definition.

**Figure 7:**
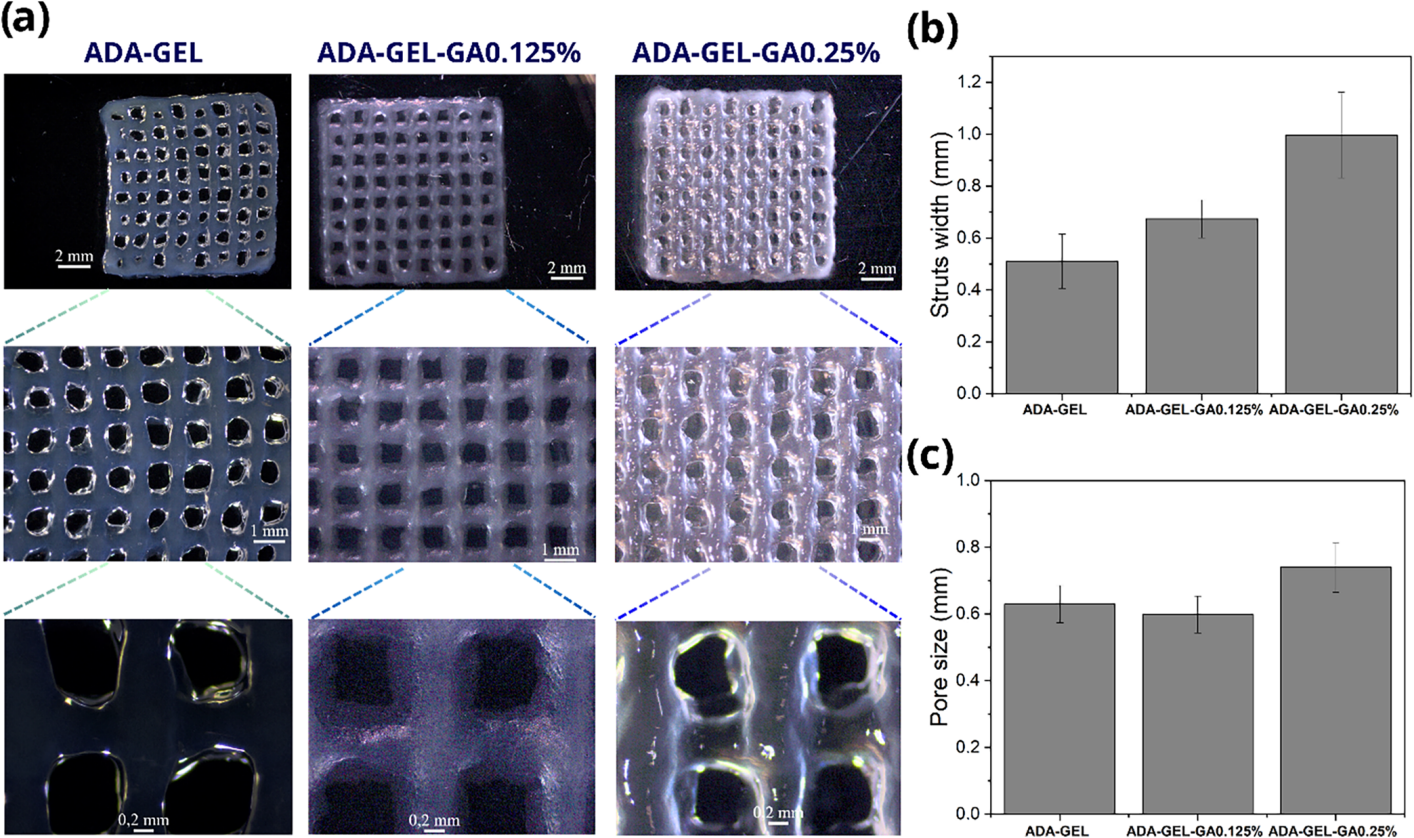
Analysis of the scaffolds (a) Optical microscopy images of 3D-printed scaffolds prepared with ADA-GEL-GA_*x*_ (*x* = 0, 0.125%, and 0.25%); (b) strut width; and (c) pore size of the scaffolds.

Strut width measurements revealed a progressive increase with higher GA concentrations, reaching approximately 1 mm for ADA-GEL-GA0.25%, Figure 7b. In contrast, pore size measurements did not show significant differences between formulations, with an average pore diameter of approximately 0.65 mm across all samples, Figure 7c. Although ADA-GEL-GA0.125% visually appeared to present slightly larger pores, the quantitative analysis indicated only subtle differences between formulations, within the experimental deviation. These results are consistent with the printability tests, in which ADA-GEL-GA0.25%, due to its lower viscosity, produced structures with increased filament spreading and less defined pore geometry.

### Biological Properties

To evaluate the biomedical potential of the materials, cell viability assays were performed on both the films and 3D-printed scaffolds (Figure 8a,b) using the preosteoblastic MC3T3-E1 cells. To further investigate the cytotoxicity of gallic acid (GA), cells were also exposed to GA alone, without the polymeric matrix, at concentrations equivalent to those present in 200 μL of each film formulation (1, 0.5, and 0.25 mg/mL). GA exposure resulted in reduced cell viability compared to the control after 1 day of incubation (Figure S7a). Fluorescence microscopy images stained with calcein AM (viable cells) and 4′,6-diamidino-2-phenylindole - DAPI (cell nuclei) revealed a progressive decrease in cell number with increasing GA concentration, (Figure S7b). These findings are consistent with previous reports indicating that GA may exhibit cytotoxic effects at higher concentrations.^54^

**Figure 8:**
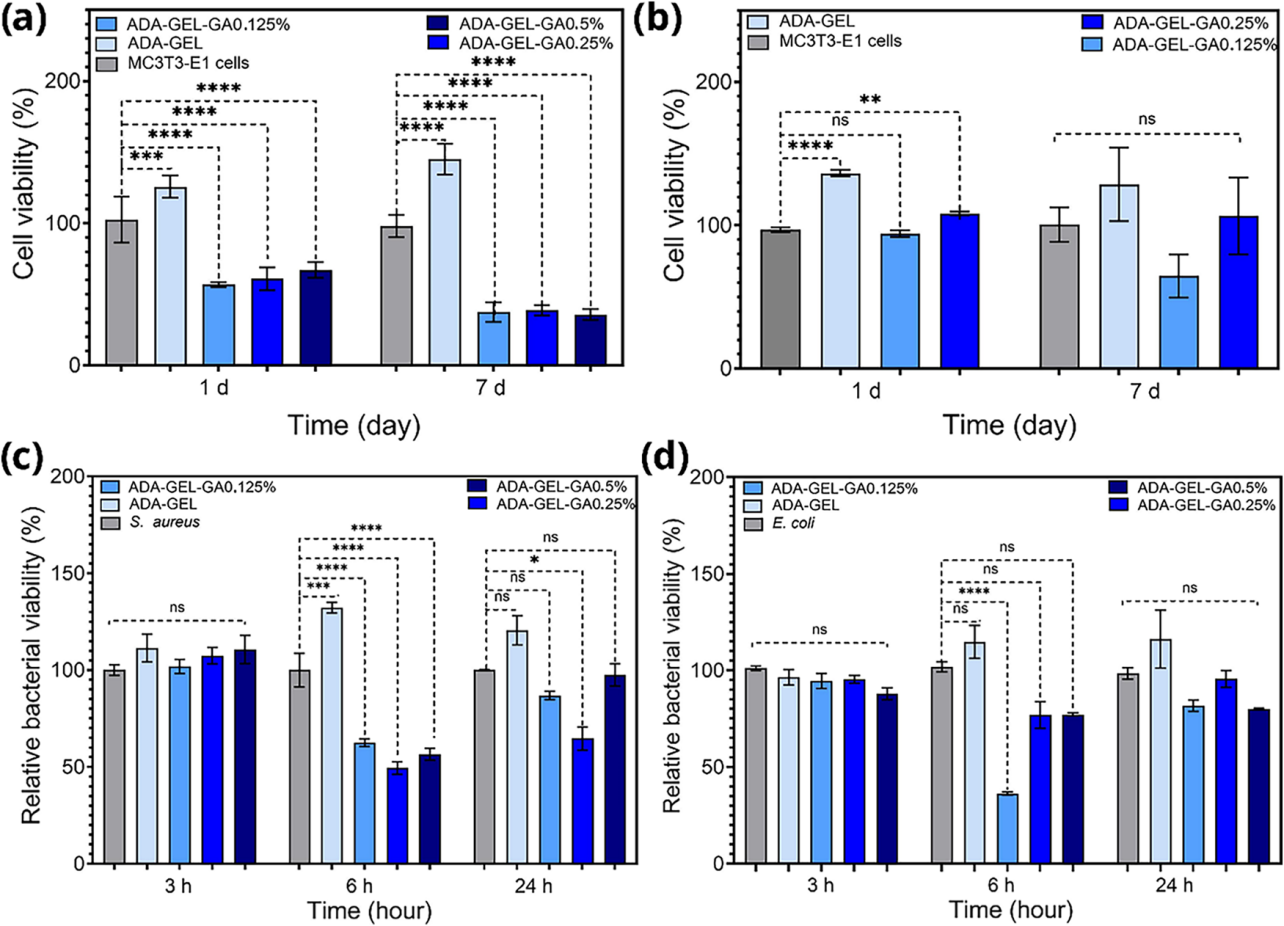
Biological assays (a) Cell viability of ADA–GEL–GA_*x*_ films (*x* = 0, 0.125%, 0.25%, and 0.5%); (b) cell viability of ADA–GEL–GA_*x*_ scaffolds (*x* = 0, 0.125%, and 0.25%); and antibacterial activity of ADA–GEL–GA_*x*_ films (*x* = 0, 0.125%, 0.25%, and 0.5%) against (c) *S. aureus* and (d) *E. coli* bacterial colonies.

In contrast to the phytotherapeutic compound alone, the GA-containing films exhibited approximately 70% cell viability on day 1, although lower than the ADA-GEL control, which promoted cell proliferation. By day 7, cell viability further decreased in the GA-loaded films, likely due to the higher concentration of gallic acid, which may exert cytotoxic effects at elevated levels. These results suggest that the polymeric matrix contributed to improved cell tolerance, likely by attenuating the cytotoxic effects associated with free GA.

This observation is consistent with the GA release data. On day 1, the released concentrations were approximately 29%, 39% and 72% for GA0.5%, GA0.25%, and GA0.125% films, respectively. By day 7, the cumulative release reached approximately 35% for GA0.5%, 55% for GA0.25%, and 88% for GA0.125%, corresponding to about 205.7 μM, 102.9 μM, and 129.4 μM of GA, respectively. According to the literature, phenolic compounds and gallic acid generally exhibit low cytotoxicity toward MC3T3-E1 cells at lower micromolar concentrations.^55,56^

However, the scaffolds, ADA–GEL–GA_*x*_, *x* = 0, 0.125% and 0.25%, showed significantly improved cytocompatibility with MC3T3-E1 cells, with cell viability exceeding 95% for GA concentrations of 0.125% and 0.25%, and up to 10% cell proliferation observed in the ADA-GEL-GA0.25% group. On day 7, viability declined to 65% in the ADA-GEL-GA0.125% scaffold, whereas ADA-GEL-GA0.25% maintained viability close to 100%. These results in the scaffolds may be attributed to the lower effective concentration of GA compared to the films due to differences in total material mass and GA distribution, which results in reduced cytotoxicity and enhanced biocompatibility. Similarly, ferulic acid incorporated into ADA–GEL scaffolds exhibited no cytotoxic effects showing cell viability close to 80%, as reported previously^25^

The antibacterial tests were performed using ADA–GEL–GA_*x*_ films, where *x* = 0, 0.125%, 0.25%, or 0.5%, against both Gram-positive (*S. aureus*) and Gram-negative (*E. coli*) bacterial colonies (Figure 8c,d). In both cases, during the first 3 h, no significant differences were observed between the ADA–GEL control and the films containing gallic acid (GA). After 6 h of incubation, an increase in the bactericidal activity of the materials was observed. For *S. aureus*, bacterial viability decreased to approximately 55% in the GA-containing films, whereas the ADA–GEL control (without GA) showed bacterial proliferation. In the case of *E. coli*, the ADA–GEL–GA0.125% film exhibited a significant reduction in bacterial growth to around 35%.

Finally, after 24 h, only the ADA–GEL–GA0.25% film exhibited a statistically significant reduction in *S. aureus* growth. Nevertheless, for both bacterial strains (*S. aureus* and *E. coli*), all ADA–GEL–GA films demonstrated enhanced antibacterial performance compared to the ADA–GEL control, with a more pronounced effect against *S. aureus*, an opportunistic resident microbiota. Similar selective behavior has been previously reported in the literature,^33,57–61^ and may be related to the absence of a robust outer membrane in Gram-positive bacteria, which makes them more susceptible to phenolic compounds, for example, through damage to the bacterial cell membrane.^5^

These results are highly promising for tissue engineering applications, particularly in bone regeneration, since *S. aureus* is one of the main pathogens responsible for osteomyelitis, a chronic bone infection that severely impairs healing. Thus, the incorporation of gallic acid effectively enhances the antimicrobial properties of the composite material. In addition, its antioxidant activity contributes to reducing inflammation in infected environments, thereby preventing bacterial proliferation and promoting tissue regeneration.

## Conclusions

In this study, different concentrations of gallic acid (GA) were successfully incorporated into ADA-GEL hydrogel. Experiments using Folin–Ciocalteu and DPPH demonstrated a high phenolic content with antioxidant activity reaching up to 96%. Swelling and degradation tests revealed similar behaviors among all formulations and gallic acid release percentages was high for lower concentrations by strong intermolecular interactions. Printability studies demonstrated that GA incorporation influenced the rheological behavior of the hydrogels, as also evidenced by the mechanical tests, while the resulting scaffolds maintained a consistent average pore size suitable for tissue engineering applications. Cellular viability assays using MC3T3-E1 preosteoblasts indicated superior performance for the scaffolds compared to films, with ADA-GEL-GA0.25% maintaining near 100% cell viability, demonstrating the biocompatibility of the scaffolds. Additionally, all films exhibited significant antibacterial activity against *S. aureus*, suggesting that these materials can prevent bacterial colonization at 6 h. Overall, the combination of antioxidant and antibacterial properties, along with printability and biocompatibility, positions ADA-GEL-GA_*x*_ hydrogels as promising candidates for advanced tissue engineering applications, including bone regeneration, where multifunctional biomaterials are required to support tissue regeneration while mitigating oxidative stress and bacterial infection.

## Materials and Methods

### Materials

Reagents used in this study include gelatin from porcine skin (Type A, GEL, gel strength 300 bloom), sodium (meta)­periodate (NaIO_4_), calcium chloride dihydrate (CaCl_2_.2H_2_O), ethylene glycol, gallic acid (3,4,5-trihydroxybenzoic acid, 97.5–102.5% by titration), glutaraldehyde, paraformaldehyde, Folin–Ciocalteu’s phenol reagent, sodium carbonate (Na_2_CO_3_), 1,1-diphenyl-2-picrylhydrazyl (DPPH), deuterated water (D_2_O) and cell counting kit-8 (WST-8) all purchased from Sigma-Aldrich. Alginate (VIVAPHARM, PH163S2) was purchased from JRS PHARMA GmbH & Co.KG. Dulbecco’s Phosphate Buffered Saline (DPBS), trypsin, Dulbecco’s Modified Eagle Medium (DMEM, 1.0 g/L d-Glucose), penicillin-streptomycin (PS), Hank’s Balanced Salt Solution (HBSS), 4′,6-diamidino-2-phenylindole (DAPI) and Calcein AM were obtained from Gibco, Thermo Fisher. Fetal bovine serum (FBS) was from Corning, Methanol and Ethanol (>99.8%) were purchased from Merck.

### Synthesis of Alginate Dialdehyde (ADA)

ADA was synthesized following the methodology reported by Karakaya et al.^19^ In this method, 50 mL of an aqueous NaIO_4_ solution (1,337 g) were slowly added to 50 mL ethanol suspension of alginate (10 g). The mixture was stirred for 6 h in a water bath under dark conditions. After this period, 10 mL of ethylene glycol was added and mixed for an additional 30 min. The ADA then sedimented, was dialyzed for 4 days, and freeze-dried.

### Synthesis of ADA-GEL-GA Hydrogel

The inks were prepared using the method described by Bider et al.^25^ Briefly, the lyophilized ADA (5%) (w/v) and GA (0, 0.25%, 0.5% or 1%) (w/v), for 3 h were completely dissolved in DPBS. A gelatin solution (7.5%) in DPBS was then prepared in a water bath at 37 °C, for 1 h, and added to ADA-GA solution in a 1:1 ratio. The mixture was stirred for exactly 10 min at 37 °C. Finally, ADA-GEL-GA hydrogel with total GA contents of 0.125%, 0.25%, or 0.5% (w/v) GA was obtained, Figure 1.

### ADA-GEL-GA Film Preparation

200 μL of ADA-GEL-GA hydrogel was added to each well of precooled cylindrical silicon molds (diameter: 12 mm, height: 2 mm) placed in Petri dishes, then cooled at 4 C for an additional 20 min. Subsequently, the molds were covered with a 0.1 M CaCl_2_ solution for 10 min, after which the molds and CaCl_2_ were removed, and the films were washed with DPBS, Figure 1.

### Nuclear Magnetic Resonance (NMR)

^1^H NMR spectra were recorded on a Varian UNMRS 400 MHz spectrometer operating at 400 MHz for proton observation, using D_2_O as the deuterated solvent. Chemical shifts (δ) were expressed in parts per million (ppm) relative to the residual solvent signal, and spectra were acquired at ambient temperature.

### Fourier-Transform Infrared Spectroscopy (FTIR)

The analysis used to identify functional groups was Fourier transform infrared spectroscopy (IRAffinity-1S, Shimadzu) with attenuated total (ATR), The spectra were collected in absorbance mode between 4000 and 400 cm^–1^ with a spectral resolution of 4 cm^–1^ and 40 spectral scans. For this, the hydrogels were freezing dried.

### Raman Spectroscopy

To improve the identification of functional groups, Raman spectra were recorded using an XploRA Plus spectrometer (Horiba) equipped with a Syncerity OE CCD detector and a 532 nm excitation laser. Measurements were performed with a 10× objective (Olympus BX43 microscope) over the 200–3300 cm^–1^ range, with a spectral resolution of 4.2 cm^–1^.

### X-ray Diffraction (XRD)

To study the type of organization of the materials, the hydrogels were freeze-dried, and an X-ray diffractometer (Rigaku, MiniFlex 600) equipped with a Cu Kα 1.2 secondary-Monochromato was used. The spectra were obtained between 5 and 50°, with a resolution of 0.02 and a scanning rate of 4 °/min.

### Thermogravimetric Analysis (TGA)

The thermal weight loss analysis of the hydrogels was performed using a Shimadzu TGA-60/60H thermogravimetric analyzer equipped with a platinum sample holder. Measurements were carried out under a synthetic air atmosphere, with a gas flow rate of 100 mL·min^–1^ and a heating rate of 10 °C·min^–1^, over a temperature range from 25 to 600 °C.

### Scanning Electron Microscopy (SEM)

To obtain the most accurate micro morphology, the films were fixed using SEM-FIX 1 solution (0.1% glutaraldehyde and 2% paraformaldehyde) and SEM-FIX 2 solution (0.3% glutaraldehyde and 3% paraformaldehyde), for 1 h in each solution. After this, they were submerged in ethanol series solutions, from 30% to 99%, and were dried using a critical point dryer (EM CPF3000, Leica). For Scanning electron microscopy (SEM), they were mounted on carbon tape and coated with a layer of gold, thickness 5.5 nm.

### Folin-Ciocalteu Assay

For measurement of total phenolic content in ADA-GEL films with different concentrations of Gallic Acid (0.125%, 0.25%, or 0.5%), the Folin-Ciocalteu assay was utilized, with modifications to the protocol.^25,62^ The films were covered with 5 mL of DPBS and incubated at 37 °C for 7 days. At specific time points (3, 24, 72, and 168 h), 1 mL of the supernatant was collected and replaced with fresh DPBS. From this 1 mL, 20 μL was mixed with 1.58 mL of deionized water and 100 μL of Folin–Ciocalteau’s phenol reagent. After 5 min, 300 μL of 20% Na_2_CO_3_ was added to the solution. The final solution was incubated for 2 h at room temperature, and the absorbance was measured at 765 nm using a ultraviolet–visible (UV–vis) spectrophotometer (Specord 40, Analytik Jena GmbH, Jena, Germany). The measurements were performed in quintuplicate. A calibration curve was generated using solutions of DPBS and gallic acid at varying concentrations (100–700 mg/L) following the same protocol as described for ADA-GEL-GA films. The blank solution consisted of pure DPBS.

### DPPH Assay

The 1,1-diphenyl-2-picrylhydrazyl (DPPH) assay was performed to evaluate the antioxidant activity of ADA-GEL-GA_*x*_ films (*x* = 0.125%, 0.25%, and 0.5%). Film samples were placed in 15 mL Falcon tubes, to which 2.5 mL of methanol was added. The tubes were kept at room temperature overnight for extraction. A 0.04 mg/mL DPPH solution was freshly prepared in methanol and stored in dark to prevent photodegradation. Then, 2.5 mL of the DPPH solution was added to each falcon tube containing the films extracts. The mixtures were incubated in the dark for 30 min. After incubation, the absorbance at 517 nm was measured in UV–Vis spectrophotometer (Specord 40, Analytik Jena GmbH, Jena, Germany). Methanol was used as the blank and the DPPH solution as the control. The measurements were performed in triplicate. The antioxidant activity (%) was calculated using the equation

1 $$antioxidant\;activity\;\%=\frac{Abs\;control-Abs\;sample}{Abs\;control}\times 100$$

### Degradation/Swelling Assay

To understand the swelling and degradation behavior, ADA-GEL-GA_*x*_ films (*x* = 0, 0.125%, 0.25%, and 0.5%) were prepared in sterile conditions. Each film and the corresponding sieve were weighed to obtain the initial weight (*W*_0_), then immersed in 8 mL of DMEM supplemented with 1% penicillin-streptomycin (PS). Samples were incubated at 37 °C in a humidified atmosphere containing 5% CO_2_ and 95% relative humidity. At predetermined time points: 30 min, 1 h, 1 h 30 min, 2 h, and on days 1, 3, 7, 14, 21, and 28, the films were weighed to determine their wet weight (*W*_t_). The DMEM medium was refreshed every 3 days to maintain physiological conditions. The measurements were performed in quintuplicate. The percentage of swelling or degradation was calculated using the following equation

2 $$\mathrm{swelling}/\mathrm{degradation}\;\%=\frac{\mathrm{Wt}}{W0}\times 100$$

### Gallic Acid Release Assay

The release profile was evaluated using ADA-GEL-GA_*x*_ films (*x* = 0.125%, 0.25%, and 0.5%) which were individually immersed in 10 mL of HBSS solution, in a quintuplicate. The samples were incubated at 37 °C in a humidified atmosphere containing 5% CO_2_ and 95% relative humidity. At predetermined time points: 0.125, 1, 3, 7, 14, 21, and 28 days, 100 μL was removed from release medium and immediately replaced with an equal volume of fresh HBSS. The Gallic Acid release was determined by measuring the absorbance at 260 nm using a UV–Vis spectrophotometer (Specord 40, Analytik Jena GmbH, Jena, Germany). A calibration curve was prepared using gallic acid standards in HBSS, ranging from 0.3905 to 12.5 mg/L. The concentration of gallic acid in each sample was quantified based on this standard curve.

### Mechanical (Stiffness) Analysis

To assess the influence of GA on the mechanical properties, ADA-GEL-GA_*x*_ films (diameter: 5 mm, height: 2 mm) were characterized using a microtester (Microtester LT, CellScale, Canada). Samples were analyzed immediately after cross-linking and subsequent washing with HBSS. The microtester cantilever consisted of a tungsten microbeam with a diameter of 0.5588 mm equipped with a 6 × 6 mm^2^ compression plate. Force-controlled cyclic compression testing was performed to investigate the mechanical deformation behavior of the hydrogels under defined loading conditions. Four consecutive compression cycles were applied using a maximum compressive force of 10,000 μN. Each cycle comprised a loading phase of 20 s, a holding phase of 2 s, an unloading/recovery phase of 20 s, and a final holding phase of 2 s. Force–displacement data were recorded continuously and converted into engineering stress and engineering strain using the initial sample geometry. Due to the viscoelastic nature of the hydrogels and to minimize initial settling and preconditioning effects, the effective compressive modulus was determined from the loading segments of the last three compression cycles. Linear regression was performed within the initial quasi-linear region of the stress–strain curves between 0.5% and 2.5% engineering strain.

### 3D Printing and Printability

ADA-GEL-GA_*x*_ hydrogels, with GA (*x* = 0.125% and 0.25%), were loaded into printing cartridges and placed in a 4 °C fridge for 20 min to improve the gelatinization and facilitate extrusion. All formulations were printed using a BioX bioprinter (CELLINK, Sweden) equipped with a cylindrical micronozzle of 250 μm in diameter (25G). The scaffold structures were printed with four layers under the following conditions: printing speed of 5 mm/s, pressure of 45 kPa, and ambient temperature. The scaffolds were cross-linked by immersion in a 0.1 M CaCl_2_ solution for 10 min and washed with DPBS to remove excess CaCl_2_ (Figure 1).

The printability tests, including the Filament Fusion Test (FFT) and the Filament Collapse Test (FCT) were performed in triplicate. For the FFT, lines were printed at standard interfilament distances (fd) of 2, 1.5, 1, 0.75, and 0.5 mm. The resolution was evaluated by calculating the fused segment ratio (fs/ft), where fs is the length of the fused segment and ft is filament thickness. Lower filament distances resulting in complete fusion were indicative of poorer resolution. The FCT was performed by printing a single filament across support pillars spaced at 16, 8, 4, 2, and 1 mm. The deflection angle of the suspended filament between pillars was measured as an indicator of structural stability.

### In Vitro Cytocompatibility Test

To analyze the cytotoxic effect, the ADA-GEL-GA_*x*_ films and scaffolds, as well as GA solutions, were synthesized under sterile conditions, and tested in triplicate. MC3T3-E1 preosteoblastic cells were cultured in DMEM supplemented with 10% of Fetal bovine serum (FBS) and 1% of penicillin-streptomycin (PS). Once cells reached 70–90% confluency in a humidified incubator at 37 °C with 5% CO_2_ and 95% relative humidity, they were detached using trypsin and washed with DPBS. For cell viability assay, the direct test was performed. Each sample was placed in a well and covered with cell suspension at a density of 1 × 10^4^ cells/sample. After allowing for initial cell attachment, 1 mL of 5% (v/v) solution of cell counting kit-8 (WST-8) was added to each well. The plates were incubated for 3 h under standard conditions, the 100 μL samples were taken and put into 96 well plates. The absorbance was checked at 450 nm using a microplate reader (PHOmo microplate reader, China). Cell viability was calculated using the following equation

3 $$\mathrm{cell}\;\mathrm{viability}\;\%=\frac{\mathrm{Abs}\;\mathrm{sample}-\mathrm{Abs}\;\mathrm{blank}}{\mathrm{Abs}\;\mathrm{ref}-\mathrm{Abs}\;\mathrm{blank}}\times 100$$

Cells cultured without samples were used as a reference, Abs ref, and the blank was WST-8 solution, Abs blank. In addition, fluorescence images of cells exposed to GA solutions were obtained using an inverted epifluorescence microscope (Axio Scope A.1, Carl Zeiss, Germany). Cell viability was evaluated using Calcein AM (staining of living cells), while nuclei were stained with DAPI. Samples were washed with HBSS between each staining step.

### Antibacterial Test

ADA-GEL-GA_*x*_ films (where *x* = 0, 0,125%, 0.25% and 0.5%) prepared under sterile conditions, in triplicate, and evaluated for antibacterial activity against *E. coli* (*E. coli*) and *S. aureus* (*S. aureus*), representing Gram-negative and Gram-positive bacteria, respectively. Initially, 10 mL of lysogeny broth (LB) medium was inoculated with each bacterial strain and incubated overnight at 37 °C. The resulting cultures were adjusted to an optical density (OD) of 0.015 at 600 nm using a spectrophotometer (GENESYS 30, Thermo Scientific, Germany). For the direct antibacterial assay, 2 mL of the adjusted bacterial suspension in LB medium was added to sterile 15 mL Falcon tubes containing the test films, 30 μL of the bacterial suspension was added to each tube. The samples were incubated at 37 °C, and bacterial growth was monitored by measuring absorbance at 600 nm at three time points: 3, 6, and 24 h, using a microplate reader (PHOmo, Anthos Mikrosysteme GmbH, Germany). The relative bacterial viability (%) was calculated using the following equation

4 $$\mathrm{relativity}\;\mathrm{bacterial}\;\mathrm{viability}\;\%=\frac{\mathrm{Abs}\;\mathrm{sample}-\mathrm{Abs}\;\mathrm{blank}}{\mathrm{Abs}\;\mathrm{ref}-\mathrm{Abs}\;\mathrm{blank}}\times 100$$

Bacteria cultured without samples were used as a reference, Abs ref, and the blank was LB medium, Abs blank.

### Statistical Analysis

Statistical analysis was performed using analysis of variance (ANOVA) to analyze the statistical differences between groups, where *p* < 0.05 = *, *p* < 0.01 = ** and *p* < 0.001 = ***.

## Supplementary Material



## Notes

X-ray diffraction (XRD); thermogravimetric analysis (TGA) of hydrogels; thermogravimetric analysis (TGA) with differential thermal analysis (DTA); calibration curve of estimation of total phenolic content by Folin & Ciocalteu′s phenol reagent; calibration curve of gallic acid; cumulative gallic acid (GA) releases (%) and cell viability with fluorescent microscopic images of GA (PDF)

## §

National University of Science and Technology Politehnica Bucharest, 060042, Bucharest, Romania
