# Supplementary material for: 3D-Printed Alginate Dialdehyde (ADA)–Gelatin (GEL) Hydrogels with Gallic Acid (GA) for Enhanced Multifunctional Properties
Source: ACS Omega. 2026 Jun 2;11(23):34442–55. doi: 10.1021/acsomega.6c02404 (PMC13280874; doi:10.1021/acsomega.6c02404)
Supplement: Supplementary file 1 [file ao6c02404_si_001.pdf]

3D-Printed alginate dialdehyde (ADA)–gelatin (GEL) hydrogels with Gallic Acid (GA) for enhanced multifunctional properties

Caroliny Oliveira Cavalcante,[a] *Hannah Sophia Kissel,*[b] *Andreea Luiza Mîrt,*[b,♦] *José Yago R. Silva,*[a] *Severino Alves Júnior,*[*a] *Aldo R. Boccaccini*[*b]

a) Departamento de Química Fundamental, Universidade Federal de Pernambuco, Cidade Universitária, 50740-560, Recife, PE, Brazil.

b) Institute of Biomaterials, Friedrich-Alexander University Erlangen-Nuremberg, 91058 Erlangen, Germany.

* Corresponding authors.

E-mail address: severino.alvesjr@ufpe.br (Severino Alves Júnior), aldo.boccaccini@fau.de (Aldo R. Boccaccini)

Supporting Information

♦ - Present address: National University of Science and Technology Politehnica Bucharest, 060042, Bucharest, Romania

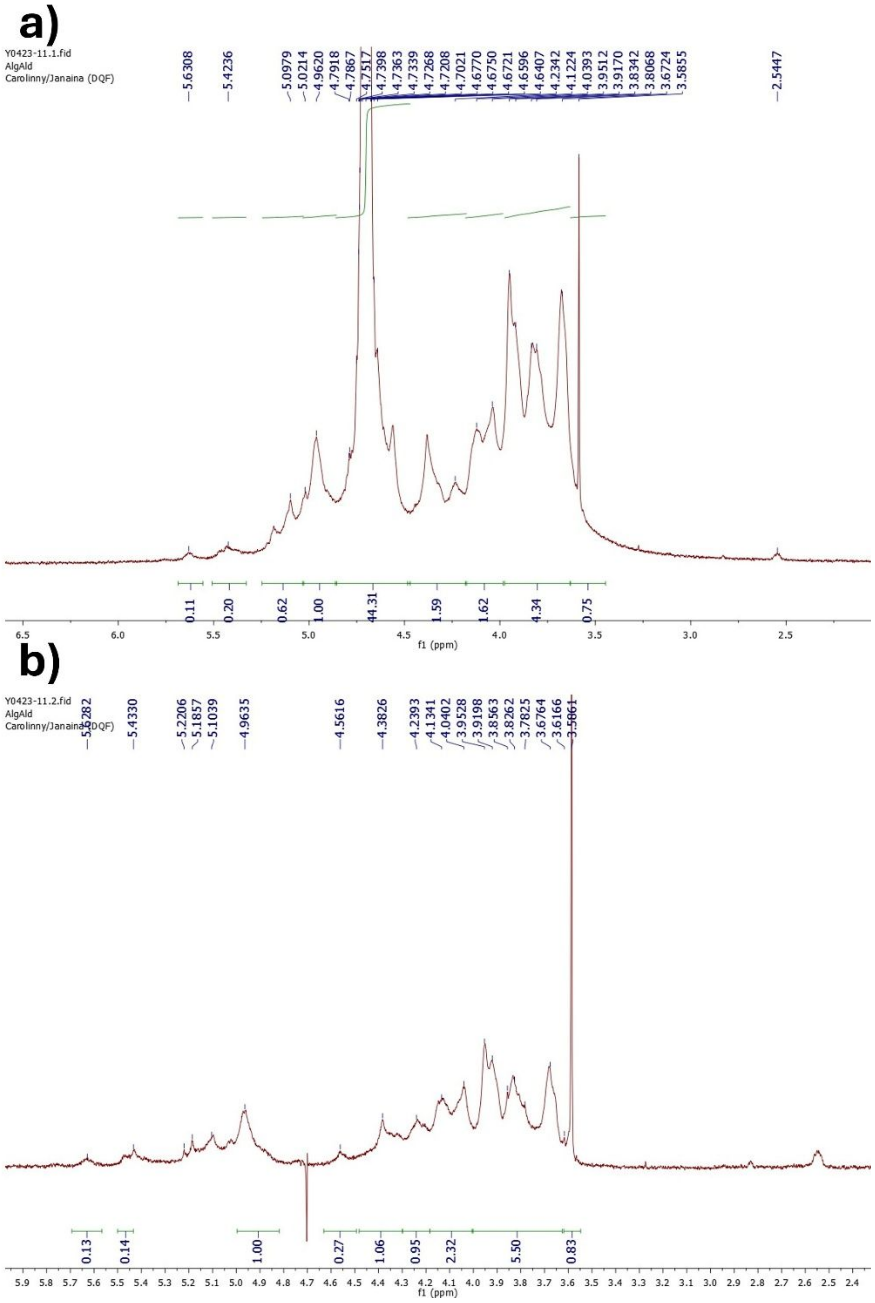

Figure S1. Proton Nuclear Magnetic Resonance (^{1}H NMR) spectra of ADA (a) in deuterated water (D_2O) and (b) with D_2O suppression

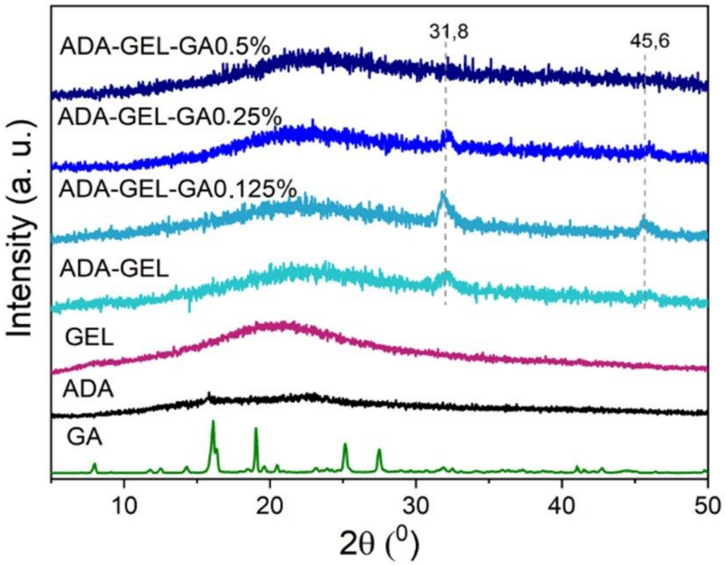

Figure S2. X-ray diffraction (XRD) of Gallic Acid (GA), alginate dialdehyde synthesis (ADA), gelatin (GEL), hydrogels (ADA-GEL, ADA-GEL-GA0.125%, ADA-GEL-GA0.25%, ADA-GEL-GA0.5%)

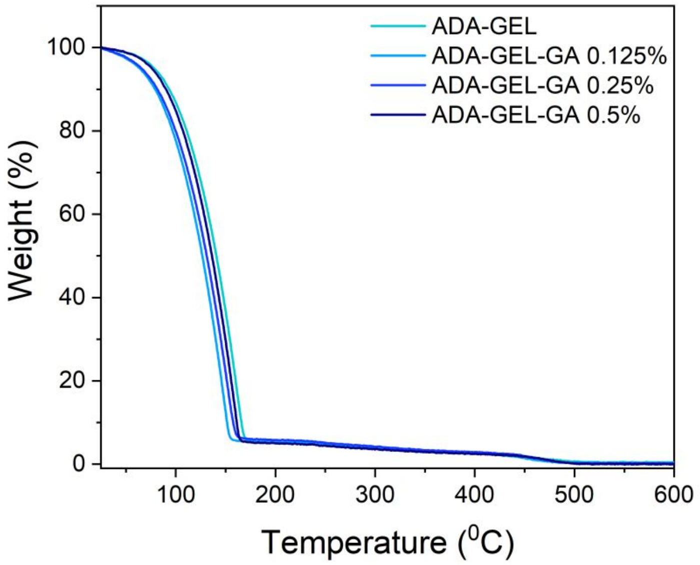

Figure S3. Thermogravimetric analysis (TGA) of hydrogels (ADA-GEL, ADA-GEL-GA0.125%, ADA-GEL-GA0.25%, ADA-GEL-GA0.5%)

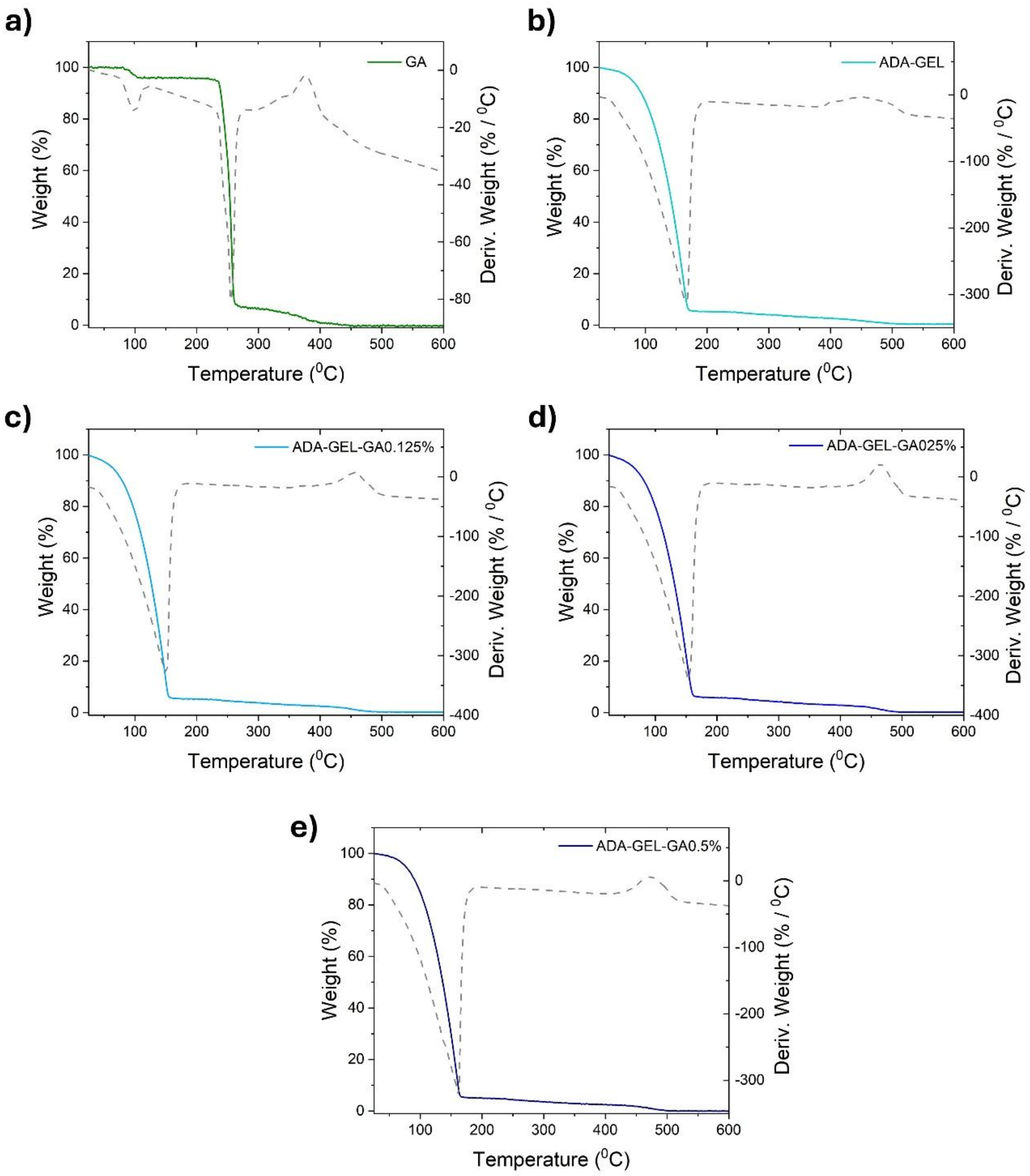

Figure S4. Thermogravimetric analysis (TGA) with Differential Thermal Analysis (DTA)of (a) Gallic Acid and hydrogels (b) ADA-GEL, (c) ADA-GEL-GA0.125%, (d) ADA-GEL-GA0.25%, (e) ADA-GEL-GA0.5%

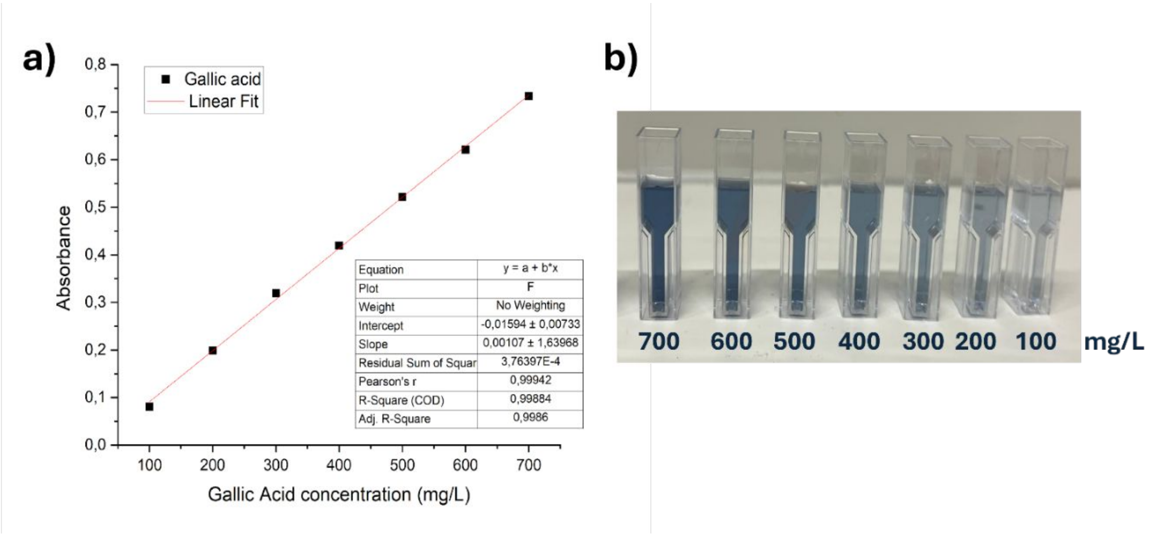

Figure S5. (a) Calibration curve of estimation of total phenolic content by Folin & Ciocalteu´s phenol reagent (b) image of cuvettes with solutions

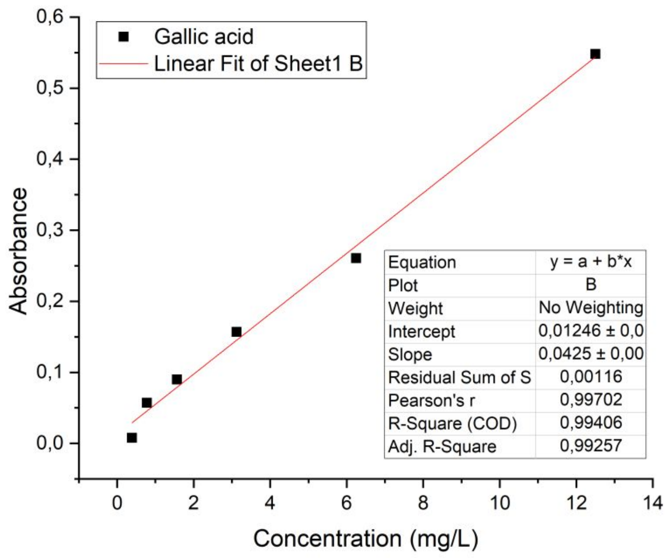

Figure S6. Calibration curve of Gallic acid

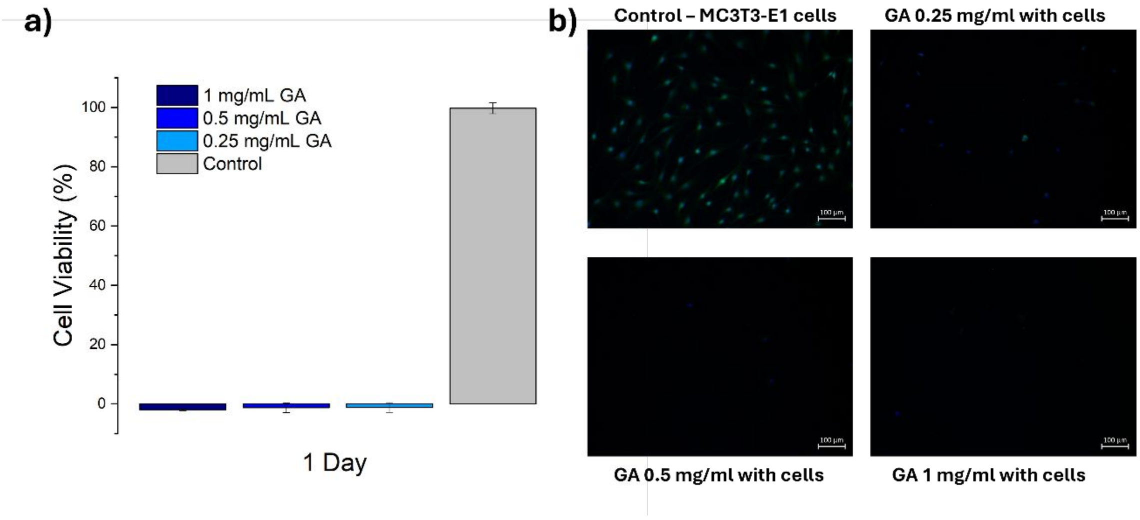

Figure S7. (a) Cell viability and (b) Fluorescent microscopic images of GA concentrations (1, 0.5 and 0.25 mg/ml) with MC3T3-E1 cells after 1 day of incubation. Green: Calcein AM and blue: DAPI.
